# Early monitoring of the human polyomavirus BK replication and sequencing analysis in a cohort of adult kidney transplant patients treated with basiliximab

**DOI:** 10.1186/1743-422X-8-407

**Published:** 2011-08-17

**Authors:** Elena Anzivino, Anna Bellizzi, Anna Paola Mitterhofer, Francesca Tinti, Mario Barile, Maria Teresa Colosimo, Daniela Fioriti, Monica Mischitelli, Fernanda Chiarini, Giancarlo Ferretti, Gloria Taliani, Valeria Pietropaolo

**Affiliations:** 1 Department of Public Health and Infectious Diseases, "Sapienza" University of Rome, Rome, Italy; 2 Department of Clinical Medicine, Nephrology and Dialysis Unit, "Sapienza" University of Rome, Rome, Italy; 3 Department of Infectious and Tropical Diseases, "Sapienza" University of Rome, Rome, Italy; 4 National Institute for Infectious Diseases Lazzaro Spallanzani, Rome, Italy; 5 Doctoral School of Oncology and Genetics, University of Siena, Siena, Italy; 6 Sbarro Institute for Cancer Research and Molecular Medicine, Center for Biotechnology, College of Science and Technology, Temple University, Philadelphia, Pennsylvania, USA

**Keywords:** BKV, BKVAN, basiliximab, Q-PCR, TCR, VP1, BKV subtype/subgroup

## Abstract

**Background:**

Nowadays, better immunosuppressors have decreased the rates of acute rejection in kidney transplantation, but have also led to the emergence of BKV-associated nephropathy (BKVAN). Therefore, we prospectively investigated BKV load in plasma and urine samples in a cohort of kidney transplants, receiving basiliximab combined with a mycophenolate mofetil-based triple immunotherapy, to evaluate the difference between BKV replication during the first 3 months post-transplantation, characterized by the non-depleting action of basiliximab, versus the second 3 months, in which the maintenance therapy acts alone. We also performed sequencing analysis to assess whether a particular BKV subtype/subgroup or transcriptional control region (TCR) variants were present.

**Methods:**

We monitored BK viruria and viremia by quantitative polymerase chain reaction (Q-PCR) at 12 hours (Tx), 1 (T1), 3 (T2) and 6 (T3) months post-transplantation among 60 kidney transplant patients. Sequencing analysis was performed by nested-PCR with specific primers for TCR and VP1 regions. Data were statistically analyzed using χ^2 ^test and Student's t-test.

**Results:**

BKV was detected at Tx in 4/60 urine and in 16/60 plasma, with median viral loads of 3,70 log GEq/mL and 3,79 log GEq/mL, respectively, followed by a significant increase of both BKV-positive transplants (32/60) and median values of viruria (5,78 log GEq/mL) and viremia (4,52 log GEq/mL) at T2. Conversely, a significantly decrease of patients with viruria and viremia (17/60) was observed at T3, together with a reduction of the median urinary and plasma viral loads (4,09 log GEq/mL and 4,00 log GEq/mL, respectively). BKV TCR sequence analysis always showed the presence of archetypal sequences, with a few single-nucleotide substitutions and one nucleotide insertion that, interestingly, were all representative of the particular subtypes/subgroups we identified by VP1 sequencing analysis: I/b-2 and IV/c-2.

**Conclusions:**

Our results confirm previous studies indicating that BKV replication may occur during the early hours after kidney transplantation, reaches the highest incidence in the third post-transplantation month and then decreases within the sixth month, maybe due to induction therapy. Moreover, it might become clinically useful whether specific BKV subtypes or rearrangements could be linked to a particular disease state in order to detect them before BKVAN onset.

## Background

Immunosuppressive strategies in renal transplantation aim to improve renal function, to prolong graft survival and to minimize the occurrence of adverse effects [1]. Standard immunosuppressive regimens in renal transplantation generally consist of calcineurin inhibitors (CNIs) [tacrolimus or ciclosporin], mycophenolate (mycophenolate mofetil [MMF] or entericcoated mycophenolate sodium), and corticosteroids (methylprednisolone or prednisolone) [2]. The addition of induction therapy with antilymphocyte antibodies or interleukin (IL)-2 receptor (IL-2Rα) antibodies, such as basiliximab (Simulect^®^; Novartis, Basel, Switzerland), to standard immunosuppressive regimens has reduced the risk of acute rejection episodes during the early post-transplant period when the risk of rejection is greatest [1,3]. Basiliximab is a recombinant chimeric IgG1 monoclonal antibody that binds specifically to the α-subunit of the IL-2Rα (also referred to as the CD25 antigen) on activated T cells, thereby inhibiting IL-2-mediated proliferation of T lymphocytes, a critical step in the cellular immune response involved in allograft rejection [2]. Basiliximab induction allows dose reduction of corticosteroids or CNIs, thereby minimizing the adverse effects associated with these co-administered agents. Moreover, the addition of basiliximab to a triple immunotherapy containing azathioprine or MMF resulted either in a significantly reduction in the incidence of biopsy-confirmed acute rejection episodes (40.4% and 42.5%, respectively) at 6 months when compared with placebo either in an increase of the IL-2Rα saturation period (36 vs 50 days and 36 vs 59 days, respectively) [2,4]. Thus, current treatment guidelines recommend the use of basiliximab as part of a CNI-based regimen for the prophylaxis of acute graft rejection in renal transplantation in adults, adolescents and children [5-8].

The introduction in clinical practice of newer, more potent immunosuppressive agents has been correlated with the higher prevalence of polyomavirus-associated nephropathy (PVAN) or, more specifically, BK polyomavirus-associated nephropathy (BKVAN) in renal transplant patients, indicating a relationship between the human polyomavirus BK (BKV) reactivation and the disruption of the immune system [9-11]. BKVAN is characterized by necrosis of proximal tubules and denudation of the basement membrane as a result of BKV lytic infection in kidney epithelial cells [12,13], but is often misdiagnosed as acute rejection or drug toxicity [14,15]. It occurs in 1% to 10% of kidney transplant recipients, usually manifesting in the first year following transplantation and leading to graft loss in 50% of infected patients within 2-3 years of follow-up [10,14].

The current recommendation includes screening for BKV reactivation with subsequent preventive reduction of immunosuppression with or without antiviral therapy [16]. Among all available diagnostic methods for BKV infection, analysis of BKV DNA load in plasma has been shown to have the highest predictive value for BKVAN with BKV load in plasma ≥ 10^4 ^copies/mL for > 3 weeks [17].

BKV infection is ubiquitous among the human population from early childhood, with a seroprevalence in adults of more than 80% [18]. After primary infection, BKV persists in a latent state in cells of several organs including kidney (the main site of BKV latency in healthy individuals), peripheral blood leukocytes, and maybe other sites such as the lung, eye, liver, and brain [9,19]. BKV belongs to the *Polyomaviridae *family, which includes non-enveloped DNA viruses with icosahedral capsids of 45 nm diameter containing small, circular double-stranded DNA genomes of approximately 5 Kb [20,21]. The viral genome is divided into early, late, and regulatory regions and encodes for at least six proteins, two from the early region [the large tumor antigen (TAg) and the small tumor antigen (tAg)] and four from the late region [the capsid proteins VP1, VP2, VP3 and the agnoprotein][21,22]. VP1, which is the major capsid protein, is present on the surface of the capsid and is responsible for receptor binding to host cells. In addition, VP1 is highly immunogenic, is the target of neutralizing antibody and is required for virion assembly and hemagglutination of red blood cells. Four distinct serotypes of BKV have been previously determined by hemagglutination inhibition, named subtypes I-IV by Jin et al. [23]. Genetic analyses of VP1 sequences have determined that the serotyping region contains a variable region of the BKV genome between nucleotides 1744 and 1812 (amino acids 61 to 83). Subtype I (further divided into four subgroups, each of which has a unique geographical distribution pattern: I/a, I/b-1, I/b-2 and I/c) is the most frequent in the normal human population worldwide (80%), subtype IV (further divided into six subgroups with their own geographical distribution pattern: IV/a-1, IV/a-2, IV/b-1, IV/b-2, IV/c-1 and IV/c-2) is prevalent in Asia and part of Europe, while subtypes II and III are infrequently detected in normal adults [24]. Several evolutionary studies using phylogenetic analysis suggest a co-migration of BKV and the human race to explain the geographical distribution patterns of BKV subtypes and subgroups [24-27].

The regulatory region contains the origin of DNA replication (O-block, 142 base-pairs) and sequences involved in transcriptional regulation of both the early and the late genes (promoter/enhancer elements), referred to as the transcriptional control region (TCR). The TCR of the proposed archetypal BK strain WW (WW TCR) has been arbitrarily divided into four transcription factor binding sequence blocks, called P (68 base-pairs), Q (39 base-pairs), R (63 base-pairs), and S (63 base-pairs). The different BKV strains display a marked heterogeneity in their TCR due to point mutations, deletions, duplications, and rearrangements in this region [28]. These rearrangements may play an important role in virus replication by increasing or decreasing the number or the affinities of host transcription factor binding sites [28,29]. BKV variants with rearranged TCR have been identified in various studies including kidney transplant recipients [30]. The BKV variants with rearranged TCR emerging as the majority species in blood of recipients with BKV-nephropathy are quite heterogeneous with insertions, deletions, and mixtures, but all seem to confer an increased early gene expression, a higher replication capacity and more pronounced cytopathology in cell culture compared to archetype WW TCR. Interestingly, the displacement of archetype TCR by rearranged TCR was not matched by a simultaneous displacement in the urine indicating that these sites represent largely independent replication compartments [30].

As mentioned above, although in the last decade better immunosuppression drugs, including basiliximab as induction therapy, have decreased the rates of acute rejection in kidney transplantation, they have also led to the emergence of BKVAN, that may occur early after kidney transplantation [16,31,32]. Therefore, the aim of our study was to prospectively investigate the BKV viral load in plasma and urine samples belonging to a cohort of 60 adult kidney transplant patients, treated with basiliximab combined with a MMF-based triple immunotherapy, during the first 6 months post-transplantation to monitor the trend of BKV viremia and viruria, focusing our attention on the difference between viral replication during the first 3 months, characterized by the immunosuppressive action of basiliximab, and the following 3 months, when the monoclonal antibody was completely removed and the maintenance therapy acted alone. We also performed sequencing analysis of all BKV positive samples to assess the presence of BKV TCR variants and sequencing analysis of the VP1 region to determine whether a particular BKV subtype/subgroup was more present in these anatomic areas.

## Methods

### Study Population

From January 2008 to December 2010, we enrolled 60 patients listed for and undergoing cadaveric or living donor kidney transplantations at the Department of Organ Transplantation and General Surgery "Paride Stefanini" of the "Umberto I" Hospital of Rome, Italy. These patients were evaluated for relevant clinical data including age, gender, weight, body mass index (BMI), etiology of end stage renal disease, hepatitis C (HCV) and B (HBV) and cytomegalovirus (CMV) serological status, diabetes, months of renal replacement therapy (RRT) and re-transplantation. We also recorded donor data of age, gender, weight, BMI, and cause of death. Graft characteristics and cold ischemia time were also scored (Additional File 1, Table S1).

All the patients received induction immunosoppressive therapy with two doses [20 mg within 2 hours prior to transplantation surgery (day 0) and 20 mg 4 days later (day 4)] of intravenous basiliximab and maintenance therapy with tacrolimus (or cyclosporine), MMF and corticosteroids. A MMF-based triple therapy induces a significant reduction of the basiliximab total body clearance (CL) [36.7 vs 18.3 mL/h], and an increase of its terminal elimination half-life (t½) [7.4 vs 11.5 days] and the period of IL-2Rα saturation (36 vs 59 days) [2]. Trough levels of immunosuppressive drugs were maintained within the therapeutic range.

Blood and urine samples for quantification of BKV load were collected at median times of 12 hours (Tx), 1 (T1), 3 (T2) and 6 (T3) months after transplantation in order to evaluate the trend of BKV viremia and viruria from basiliximab administration (Tx-T1) to its complete removal (T2), and the following 3 months of immunosuppressive action of maintenance therapy (T3). BKV DNA copies were measured by Q-PCR. At the same time we monitored blood count, urea, creatinine, serum sodium, and potassium levels. Renal biopsies were taken when acute rejection was suspected.

An informed consent form was signed by each patient at the time of collection.

The study was approved by the Ethics Committee of the Faculty of Medicine.

### Clinical Specimens Collection and Processing

This study was carried out on a total of 240 blood samples and 240 urine samples, collected from a cohort of 60 adult renal allograft recipients at the different times after transplantation as mentioned above.

DNA for molecular analysis was extracted from 1 ml of each urine sample, collected without preservatives, using the DNeasy^® ^Blood & Tissue Kit (QIAGEN, S.p.A, Italy) according to the manufacturer's instructions and stored at -20°C until use.

Blood samples, collected in 4-mL Vacuntainer^® ^tubes containing EDTA (BD Becton Dickinson S.p.A, Italia), were centrifuged at 1,376 g/sec for 10 minutes and DNA was extracted from 200 μL of plasma using the DNeasy^® ^Blood & Tissue Kit (QIAGEN, S.p.A, Milan, Italy) and stored at -20°C until use. Peripheral blood mononuclear cells (PBMCs) were isolated from whole blood using the standard Ficoll Hypaque density gradient centrifugation technique [33]. PBMCs DNA extraction was performed on 10^6 ^cells by the QIAmp^® ^DNA Blood Kit (QIAGEN S.p.A, Milan, Italy) according to the manufacturer's instructions and stored at -20°C until use.

DNA yield of all samples was determined by measuring its concentration in the eluate by absorbance at 260 nm and then 0.1-1 μg of total DNA was directly used for PCR amplification assays.

### Q-PCR for BKV

DNA samples extracted from all urine and plasma collected in this study were analyzed using the BKV Q-PCR Alert Kit (Nanogen Advanced Diagnostics S.r.l., Italy), a quantitative amplification assay designed for the diagnosis and monitoring of BKV infection in DNA samples extracted from plasma collected in EDTA and urine collected without preservatives. This technique, based on TaqMan-MGB^® ^(Minor Groove Binder) technology, was performed using a 7300 Real Time PCR System (AB Applied Biosystems, Foster City, CA). The amplification reaction is carried out both for a region of the gene that encodes BKV TAg and for the promoter region and 5'UTR of the human beta globin (β-globin) gene. β-globin gene is amplified simultaneously with the target sequence to verify successful DNA isolation and exclude false-negative results. PCR amplifications were run in a reaction volume of 20 μL containing 5 μL (50 ng/μl) of total purified DNA, BKV Q-PCR Alert AmpliMIX (specific primer oligonucleotides mixture), BKV Q-PCR Alert AmpliPROBE (mixture of fluorescent probes labelled with FAM/MGB-NFQ specific for BKV DNA and fluorescent probes labelled with VIC/MGB-NFQ specific for human β-globin gene, the internal suitability test of the sample) and Q-PCR Alert AmpliMASTER (optimized reagent mixture with Uracil-N-glycosidase (UNG), an enzyme to inactivate contamination from amplification product, and ROX fluorophore, as the passive reference for the normalisation of the fluorescence). Thermal cycling was carried out according to the following steps: an initial denaturation at 95°C for 10 min, followed by 45 cycles of 95°C for 15 sec and 60°C for 1 min, at the end of which the fluorescence was read. Amplification data were analyzed with software provided by the manufacturer. The standard curve for this quantitative amplification assay is obtained using the BKV Q-PCR Standard, four stabilized serial dilutions at known titre (range: 10^2^-10^5 ^plasmid copies) of a plasmid containing part of the BKV genome region encoding for TAg. All samples were tested in triplicate and the number of viral copies in each sample was calculated from the standard curve. The Q-PCR results for urine and plasma specimens were expressed as genome equivalents of viral DNA per milliliter (GEq/mL) of sample. Standard precautions designed to prevent contamination during Q-PCR were followed and a No Template Control (NTC) lane was included in each run. The assay detected about 10 molecules of the target sequence in 5 μL DNA.

### PCR for BKV TCR

All BKV-positive specimens were further amplified by a nested-PCR using two sets of primers annealing to invariant regions flanking the BKV TCR. The first amplification round was carried out using BKTT1, 5'-AAG GTC CAT GAG CTC CAT GGA TTC TTC C-3' (nucleotides 5106-5133) as forward primer, and BKTT2, 5'-CTA GGT CCC CCA AAA GTG CTA GAG CAG C-3' (nucleotides 657-630) as reverse primer, that generated a 748 bp PCR product after 40 amplification cycles [34]. The following amplification round, instead, was carried out using the forward primer BK-1, 5'-GGC CTC AGA AAA AGC CTC CAC ACC CTT ACT ACT TGA-3' (nucleotides 50-85) and the reverse primer BK-2, 5'-CTT GTC GTG ACA GCT GGC GCA GAA C-3' (nucleotides 415-391) that amplified an inner portion of the first-round PCR product, generating a fragment of 366 bp in length after 29 amplification cycles [35]. The two amplifications were performed in a total volume of 50 μl, containing 10 pmol of each primer, 0.2 mM dNTPs, 1.5 mM MgCl_2_, and 2U Bio*Taq *DNA polymerase with an appropriate reaction buffer (Tris-HCl 100 mM, pH 8.3, KCl 500 mM). In the first step, 5 μl (50 ng/μl) of purified DNA was added to the PCR mixture and, in the second step, 2,5 μl of template. PCR amplifications were carried out in a GeneAmp^® ^PCR System 9700 (AB Applied Biosystems, Foster City, CA). All assays included positive (recombinant pGem-1 plasmid DNA containing the complete BKV genome, cloned as EcoR1 fragments) and negative (PCR mixture plus pure water) controls to exclude false-positive and false-negative results. PCR products were detected by electrophoresis on an ethidium bromide-stained 2% agarose gel.

### PCR for BKV VP1 region

In order to amplify the genomic region corresponding to VP1 of BK virus, a nested-PCR with two different sets of primers was performed. The first amplification round was carried out using the forward primer VP1-7, 5'-ATC AAA GAA CTG CTC CTC AAT-3' (nucleotides 1480-1500) and the reverse primer VP1-2R, 5'-GCA CTC CCT GCA TTT CCA AGG G-3' (nucleotides 2038-2059) that generated a 579 bp PCR product after 35 amplification cycles [36]. The following amplification round, instead, was conducted using 327-1, 5'-CAA GTG CCA AAA CTA CTA AT-3' (nucleotides 1630-1649) as forward primer, and 327-2, 5'-TGC ATG AAG GTT AAG CAT GC-3' (nucleotides 1956-1937) as reverse primer, designed to amplify an inner portion of the first-round PCR product, generating a fragment of 327 bp in length after 35 amplification cycles [23]. The two amplifications were performed in a total volume of 50 μl, containing 10 pmol of each primer, 0.2 mM dNTPs, 1.5 mM MgCl_2_, and 2U Bio*Taq *DNA polymerase with an appropriate reaction buffer (Tris-HCl 100 mM, pH 8.3, KCl 500 mM). In the first step, 5 μl (50 ng/μl) of purified DNA was added to the PCR mixture and, in the second step, 2,5 μl of template. PCR amplifications were carried out in a GeneAmp^® ^PCR System 9700 (AB Applied Biosystems, Foster City, CA). Positive (BKV DNA) and negative (pure water) controls were set up in parallel to exclude false-negative and contamination. Amplified fragments were detected by ethidium bromide staining after electrophoresis on 2% agarose gel.

### Sequencing of BKV TCR and VP1 regions

PCR products corresponding to the TCR and VP1 regions were purified before sequencing to remove the excess of primers with QIAquick^® ^PCR purification kit, according to QIAGEN protocol [37]. DNA sequencing was performed by automatic DNA sequencer (Applied Biosystem, mod. 370A) according to the manufacturer's specifications (Amplicycle Kit, Applied Biosystem). Sequences were organised and analyzed by homology analysis using the Genetic Computer Group sequence analysis software package. In particular, all sequences obtained from the amplification of the TCR region were compared to the TCR sequence of the BKV archetype strain WW (GenBank accession no. AB211371), whereas those obtained from the amplification of the VP1 region were classified into known subtypes/subgroups analysing the single nucleotide polymorphisms (SNPs) within the amplified VP1 region. To detect the SNPs, by which we have sub-classified our isolates, we aligned 32 complete VP1 subtype-IV DNA sequences, divided into six subgroups, and 13 complete VP1 subtype-I DNA sequences, divided into four subgroups. We also aligned the complete VP1 gene sequences of 3 subtype II and 2 VP1 DNA sequences belonging to subtype III in order to create a consensus sequence for these subtypes (Additional File 2, Table S2). Sequence alignments were performed with ClustalW2 at the EMBL-EBI website using default parameters [38]. Sequences were numbered using BKV Dunlop as reference strain (GenBank accession no. NC_001538) following the system of Seif et al., in which nucleotide position 1 is the TCR position next to the start codon of TAg [39].

### Statistical analysis

Data were summarized as medians and ranges or as mean, as appropriate. If Z test indicated a non-normal distribution, we used non-parametric test such as Mann-Whitney U tests and Kruskal-Wallis tests. Categorical data were analyzed by using χ^2 ^test and Student's t-test. *P *values < 0.05 were considered statistically significant.

## Results

### Quantification of BKV load in urine and in plasma specimens

The Q-PCR analysis highlighted the presence of BKV in 63/240 urine samples and in 85/240 plasma samples collected from the 60 renal transplant patients at different times after transplantation (Tx, T1, T2 and T3). Tx-T2 correspond to the pharmacokinetics phase of basiliximab action from its administration (Tx-T1) to its complete elimination (T2), whereas T3 corresponds to the following 3 months of maintenance therapy alone (T3). Additional File 3, Table S3 shows in detail the BKV positivity distributed among the various types of samples analyzed in our study. In particular, BKV DNA was found in 4/60 (6%) urine samples and in 16/60 (26%) plasma samples at Tx, and therefore after the first 20 mg dose of basiliximab administration. Monitoring of BKV replication in the following 6 months showed a gradual significant increase of patients with viruria when we compared samples collected at T1 (about 1 month after the second dose of basiliximab injection) to those collected at T2, which corresponds to the maximum period of IL-2Rα saturation (59 days from the first dose). In fact, at T1, 10/60 (16%) urine were positive to viral genome whereas, at T2, urine positive raised to 32/60 (53%) (*P *< 0.05). Similarly for viremia, we found 20/60 (33%) BKV-positive blood samples at T1, whereas positive specimens were 32/60 (53%) at T2 (*P *< 0.05). Conversely, a significantly reduction of individuals with viruria and viremia (17/60; 28%) was detected at T3 (*P *< 0.05). The same trend was observed when we compared the median viral loads obtained at different times of the follow-up. In fact, urinary and plasma BKV loads significantly increased up to reach the values of 5,78 log GEq/mL and 4,52 log GEq/mL at T2 (*P *< 0.05), respectively. On the other hand, a significantly decreasing was observed 3 months after the IL-2Rα saturation phase ending (4,09 log GEq/mL in urine and 4,00 log GEq/mL in plasma) (*P *< 0.05), when the immunosuppression was exclusively due to the maintenance therapy.

Finally, there was no significant association with detection of BKV in plasma and/or urine among recipients' characteristics (age, gender, time of dialysis, etiology of renal failure and immunosuppressive therapy); donor parameters (age, gender, weight, BMI and cause of death), or graft feature (score and ischemic time) (*P *> 0.05) (data not shown).

### PCR and sequencing analysis of BKV TCR sequences

**Figure 1:**
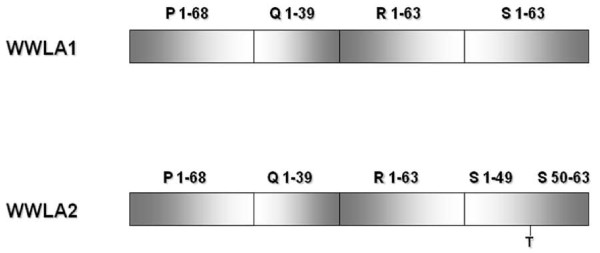
**Schematic representation of the BKV TCR variants detected in urine and in blood specimens**. The four blocks (P, Q, R and S) commonly used to denote archetypal TCRs [11] are indicated by rectangles. Above each colored rectangle is a letter confirming the name of the block, followed by a pair of numbers to indicate the range of nucleotides present. The S block of the WWLA2 archetype has one nucleotide insertion represented by a T below the line between nucleotide positions 49 and 50.

**Figure 2:**
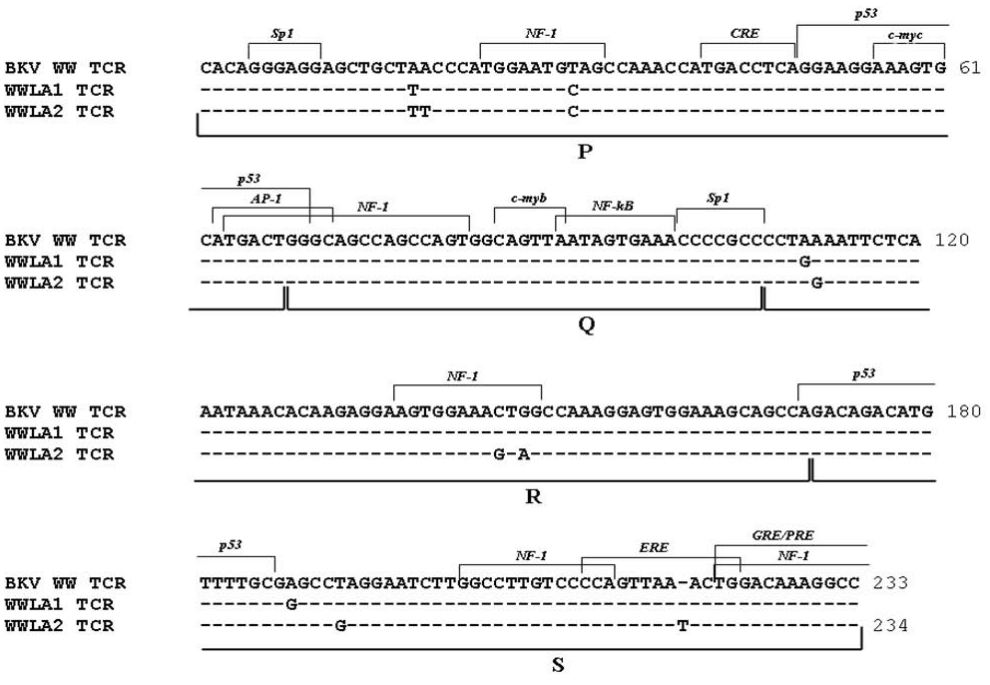
**Alignment of the WWLA1 and WWLA2 TCR sequences with BKV archetype WW strain TCR sequence**. The WW TCR is shown at the top of the figure while the nucleotide sequences of the WWLA1 and WWLA2 variants, determined directly from urine and blood samples of our study group, are shown below in relation to the WW TCR (GenBank accession no. AB211371), with similar nucleotides indicates by hyphens. Blocks (P, Q, R and S) commonly used to denote archetypal TCRs [11] are indicated under the TCR sequences, while proven and putative binding sites for transcription factors are illustrated above the WW TCR sequence.

All specimens resulted positive to viral DNA by Q-PCR assay as well as the PBMCs isolated from blood samples with viremia were amplified by nested-PCR and sequenced, successively, for BKV TCR to investigate the presence of possible rearrangements within this region. The TCR sequence derived from the total DNA of each individual clinical sample was compared to the TCR sequence of BKV archetype strain WW, available on GenBank, by using the convention described in the review by Moens et al. [40]. Specifically, the 233 nucleotides of the archetype WW TCR sequence was arbitrarily divided into four blocks (P, Q, R, and S) with lengths of 68, 39, 63, and 63 nt, respectively, to more easily describe the major rearrangements within the TCR. Homology analysis evidenced that 148 out of 233 BKV TCR analyzed sequences (63 urine, 85 plasma and 85 PBMCs) showed a TCR identical to the archetypal BKV control region (designated WW-like archetype 1 TCR or WWLA1), consisting of a single complete copy of the four blocks arranged in the correct order (Figure 1), with the occurrence of a few point mutations at nucleotide positions 18 and 31 within P block, 4 within R block and 18 within S block (Figure 2). These point mutations did not involve any cellular transcriptional factors binding sites, except for the single-nucleotide substitution at position 31 within P block involving the nuclear factor-1 (NF-1) binding site (Figure 2). For the remaining 85 analyzed sequences, obtained from 25 urine, 30 plasma and 30 PBMCs samples, a slightly altered TCR archetype, consisting of a single complete copy of the P, Q, R and S blocks arranged in the correct order, the last of which characterized by the insertion of a single thymidine nucleotide between nucleotide positions 49 and 50, was isolated (Figure 1). This archetype-like structure has been designated WW-like archetype 2 TCR (WWLA2) to distinguish it from the WWLA1 archetype. When compared with the TCR nucleotide sequences of the BKV archetype strain WW, various nucleotide substitutions have been identified in the different sequences at nucleotide positions: 18, 19 and 31 within P block, 5, 38 and 40 within R block and 22 within S block (Figure 2). The nucleotide substitutions at positions 31 within P block and 38 and 40 within R block involved two binding sites for the transcription factor NF-1, whereas the thymidine insertion between nucleotide positions 49 and 50 within the S block interested the estrogen response element (ERE) (Figure 2).

### PCR and sequencing analysis of BKV VP1 region

An additional specific nested PCR was undertaken to detect the presence of VP1 region in urine and PBMCs samples collected from all BKV-positive renal transplant patients. The 327 bp VP1 gene sequence was amplified in all specimens of our patient group (63 urine and 85 PBMCs). The amplified PCR products from all BKV VP1-positive individuals were then sequenced in order to classify each BKV strain into the corresponding subtype/subgroup analysing the SNPs within the amplified VP1 region. Alignment of 13 complete VP1 subtype I and 32 subtype IV DNA sequences, divided into the corresponding subgroups, identified seven positions (nucleotides 1687, 1698, 1809,1860, 1887, 1908 and 1923) showing SNPs within the 327 bp typing region among subtypes I subgroups and six positions (nucleotides 1731, 1794, 1851, 1860, 1905 and 1926) among subtypes IV subgroups. Based on these SNPs and on the consensus sequences for subtypes II and III, obtained aligning the complete VP1 gene sequences of 3 subtype II and 2 subtype III, subtype I/subgroup b-2 was detected in 38 out of 63 urine samples (60%) and in 55 out of 85 PBMCs samples (65%), respectively, while subtype IV/subgroup c-2 was detected in 25 urine (40%) and in 30 PBMCs specimens (35%). Subtype II and III were never detected (data not shown).

## Discussion

In the last 50 years, solid organ transplantation has been established as not only a key life saving procedure of terminal organ failure, but also as the treatment of choice with respect to patients' quality of life. In addition to better procurement and surgical procedures, much of the success of solid organ transplantation stems from improving the control of acute immune reactions across HLA-mismatches through the establishment of new efficient immunosuppressive protocols, which have efficiently reduced immune injury and acute and chronic loss of function [31]. Thus, recent regimens for the prevention of acute organ rejection in both pediatric and adult transplant patients are based on a combination of drugs such as CNIs, anti-proliferative agents and steroids, that often are supplemented by induction therapy with non-/depleting antibodies such as basiliximab, a chimeric monoclonal anti-CD25-antibody that binds to the α-peptide chain of the IL-2Rα on the surface of the antigen-activated T lymphocytes and monocytes/macrophages [2,41]. In the most recent decade, however, the declining rates of rejection episodes after kidney transplantation has no longer been translated into similarly improved rates of graft survival since better immunosuppression drugs, including basiliximab as induction therapy, have also led to the emergence of BKVAN, a major cause of renal failure early after kidney transplantation [31]. Therefore, the aim of our study was to prospectively investigate BKV load in plasma and urine samples belonging to a cohort of 60 adult renal transplant patients, treated with basiliximab and a MMF-based triple immunotherapy, to evaluate the difference between BKV replication during the first 3 months post-transplantation, characterized by the non-depleting action of basiliximab, versus the second 3 months, in which the induction agent is not longer detectable and the maintenance therapy acts alone. In addition we performed sequencing analysis of all BKV-positive samples to assess whether a particular BKV subtype/subgroup or TCR variants was present in these anatomic areas.

The Q-PCR analysis highlighted a significant gradual increase of both the total number of BKV-positive patients and the median values of viremia and viruria in the cohort of kidney transplants during the first month post-transplantation with a pick in the third month. Conversely, a significant decrease of the number of individuals with viruria and viremia was observed at 6 months, along with a reduction of the median urinary and plasma viral loads. Moreover, BK viremia or viruria did not cause renal dysfunction since graft function remained stable in all patients during the follow-up period.

These results could be explained either with the pharmacodynamic properties of basiliximab either with the change of pharmacokinetic parameters when the antibody was administered in combination with a dual- or triple-immunotherapy. As mentioned above, basiliximab binds specifically to IL-2Rα on CD3^+ ^and CD4^+^CD25^+ ^cells and competitively inhibits IL-2 binding to the receptor, thereby inhibiting IL-2-mediated proliferation of T-cells, a critical step in the cellular immune response involved in allograft rejection [2]. In fact, it has been demonstrated that basiliximab reduces the percentage of T-cells expressing IL-2Rα in renal transplant recipients from pre-treatment values of 18-44% to < 3% following receptor saturation [2]. Complete saturation of IL-2Rα on circulating CD4^+^CD25^+ ^T-cells in renal transplant recipients occurs at serum concentrations of basiliximab > 0.2 mg/mL already 1 day after administration at the recommended adult dosage of intravenous basiliximab and usually lasts for up to 4-6 weeks [2,41]. When basiliximab serum concentrations fall below the 0.2 mg/mL threshold, approximately 30 days after, the number of T-cells expressing the CD25 antigen returns to pre-therapy levels in 1-2 weeks [2,41]. It has been demonstrated that in patients receiving a triple immunotherapy with the addition of MMF, as were the patients of this study, the clearance of basiliximab is significantly reduced resulting in a prolonged duration of CD25 saturation (59 days) [42]. Basiliximab pharmacokinetic properties reflect BKV replication trend: in fact, an increase of BKV replication was observed in our patients during the first 3 months post-transplantation. Our findings are supported by Koukulaki and colleagues [43], who demonstrated that the highest incidence of BK viremia and viruria, observed in the third post-transplantation month, may be related to immunosuppression induction, along with corticosteroid doses and/or calcineurin inhibitors therapeutic trough levels, confirming previously published studies in Europe and the United States [44,45]. Moreover, the long-term follow up showed that BKV replication decreased significantly after the third post-transplant month and even transient viremia or viruria did not have an impact on renal function [43].

However, we cannot exclude that the early post-transplant viremia detected in our patients was due to other conditions, probably related to graft or recipient features, although we did not find any relation between BKV infections and recipient, donor or graft characteristics. It could be also related to the BKV serological status of both donors and recipients before transplantation, but these data were not evaluated in this study.

The natural history of BKV infection and reactivation in transplant recipients is poorly understood. However, highly effective immunosuppressive treatments are probably the main cause for the virus ability to replicate freely [46]. Early observations in renal transplant patients with BKVAN revealed frequent rearrangements of the TCR genomic region in BKV isolates recovered from various body compartments, including the urogenital compartment (urine, kidney biopsy specimens) and circulatory compartment (blood, serum, plasma) [28]. Duplications, deletions, and/or modifications that arise during reactivation of BKV may affect regulatory protein binding motifs in the region encompassing the TCR and the origin of replication [28,47]. These rearrangements may also allow the virus to adapt to ongoing changes within the host cell environment by increasing or decreasing the number or the affinities of host transcription factor binding sites [48]. Specific TCR rearrangements in urine and/or blood might become the hallmark of a high risk for progression into BKVAN if they can be linked to biological activities [28]. Therefore, we explored the structure of BKV TCR variants in urine and blood samples taken from the 60 renal transplant patients enrolled in this study to determine whether there is any association between TCR variability and the risk of BKV nephropathy onset, since an apparent association between the presence of rearranged TCR variants and BKV nephropathy was found [49]. Fortunately, no one of our recipients developed BKVAN during the first 6 months of follow-up post-transplantation and we always found archetypal TCR sequences in both urogenital and circulatory compartment. However, when we compared the 233 TCR sequences obtained from all BKV-positive clinical specimens, included those isolated from PBMCs of blood samples with viremia, to the TCR sequence of the BKV archetype strain WW, a few single-nucleotide substitutions were found. In particular 148/233 BKV TCR analyzed sequences (63 urine, 85 plasma and 85 PBMCs), designated WWLA1, showed a few point mutations at nucleotide positions 18 and 31 within P block (the latter of which involving the binding site for the transcription factor NF-1), 4 within R block and 18 within S block, whereas the remaining 85 (25 urine, 30 plasma and 30 PBMCs), named WWLA2, were characterized by various nucleotide substitutions at nucleotide positions 18, 19 and 31 within P block, 5, 38 and 40 within R block and 22 within S block, and by the insertion of a single thymidine nucleotide between nucleotide positions 49 and 50 within the S block. The point mutations at nucleotide positions 31 within P block and 38 and 40 within R block involved two NF-1 binding sites, whereas the thymidine insertion between nucleotides 49 and 50 within the S block interested the ERE binding site. It could be exciting to verify how these point mutations we detected might affect the *in vitro *viral replication.

Interestingly, the point mutations detected in all TCR analyzed sequences were representative of the particular subtypes/subgroups we identified by sequencing analysis of BKV VP1 region, as demonstrated by Yogo et al. [50]. In fact, the nucleotide substitutions detected in the WWLA1 archetype sequence corresponded to those found in the consensus TCR sequence for subtype I/subgroup b-2, when it was compared to the prototypic TCR sequence defined by Yogo and colleagues [50], whereas the various nucleotide substitutions and the single-nucleotide insertion identified in the WWLA2 archetype were typical of the subtype IV/subgroup c. Therefore, since the TCR of each subtype or subgroup of BKV has a unique set of nucleotide substitutions and single-nucleotide insertions (or deletions), the archetypal TCRs detected in clinical samples should be evaluated with reference to all the subtype- and subgroup-specific TCRs and not with the TCR of the WW strain, belonging to subtype I/subgroup b-1, that is commonly used to represent the archetypal TCR of BKV [50].

## Conclusions

In conclusion, our results confirm previously published studies indicating that BKV replication may occur during the early hours after kidney transplantation, reaches the highest incidence in the third post-transplantation month and then decreases significantly within the sixth month [43]. Moreover, monitoring of BKV viremia and viruria allows to identify renal transplant patients that could be at risk of BKVAN, since viral re-activation may occur at any time-point, although it is more likely to begin in the early period post-transplantation, including the first hours post-transplantation, maybe as a consequence of the induction therapy. Finally, in association with viral load determination, it might become clinically useful whether specific BKV subtypes or rearrangements could be linked to a particular disease state in order to develop specific test to detect them before BKVAN onset, as suggested by some Authors [29,51]. However larger patient populations and longer follow-up are necessary.

## Competing interests

The authors declare that they have no competing interests.

## Authors' contributions

EA, AB, APM, FT, MB, MTC, DF, MM, FC, GF, GT and VP conceived of the study, and participated in its design and coordination. All authors read and approved the final manuscript.

## Supplementary Material

Additional file 1 **Characteristics of recipients and donors enrolled in this study and graft conditions**. Table S1 includes the characteristics of recipients and donors enrolled in this study and graft conditions. Click here for file

Additional file 2 **BKV isolates analysed in this study**. Table S2 includes all the BKV isolates analyzed in this study. Click here for file

Additional file 3 **BKV detection in plasma and urine with relative viral load in recipients at different times**. Table S3 shows the BK viral loads detected in plasma and urine of renal transplant recipients. Click here for file
