# Supplementary material for: Early monitoring of the human polyomavirus BK replication and sequencing analysis in a cohort of adult kidney transplant patients treated with basiliximab
Source: Virol J. 2011 Aug 17;8:407. doi: 10.1186/1743-422X-8-407 (PMC3179958; doi:10.1186/1743-422X-8-407)
Supplement: Additional file 1 — Characteristics of recipients and donors enrolled in this study and graft conditions. Table S1 includes the characteristics of recipients and donors enrolled in this study and graft conditions. [file 1743-422X-8-407-S1.DOC]

Table 1. *Characteristic of recipients and donors enrolled in this study and graft conditions.*

| *Characteristics* | *All patients* (n=60) |
| --- | --- |
| Recipient  Age, years (median; range) | 47 (26-71) |
| Weight, Kg (median; range) | 68 (48-97) |
| BMI (median; range) | 24 (18-31) |
| Gender M/F (n) | 38/22 |
| *Causes of end-stage renal disease (n)* | |
| Chronic glomerulonephritis | 32/60 |
| Polycystic kidney disease, dysplasia | 14/60 |
| Congenital disease | 5/60 |
| Sclerosis | 6/60 |
| Kideny stones | 2/60 |
| Infection (TBC) | 1/60 |
| HBV HBsAg (n) | 2/60 |
| HCV infection (n) | 1/60 |
| CMV IgG positivity (n) | 26/60 |
| Diabetes (n) | 1/60 |
| Retransplant (n) | 1/60 |
| *Dialysis (n)* | |
| No dialysis | 4/60 |
| Dialysis | 56/60 |
| RRT duration, months (mean ± SD) | 41.9 ± 34.3 |
| Donor | |
| Type (deceased/living) | 59/1 |
| Age, years (median; range) | 51 (36-67) |
| Gender M/F (n) | 25/35 |
| Weight, kg (median; range) | 72 (68-76) |
| BMI (median; range) | 25 (18-32) |
| *Cause of death (n)* | |
| Subarachnoid haemorrhage | 43/60 |
| Polytrauma | 10/60 |
| Postischemic | 7/60 |
| Graft, n (%) | |
| *Score* | |
| 1 | 3/17 |
| 2 | 6/17 |
| 3 | 5/17 |
| 4 | 3/17 |
| Cold ischemia time, hours (mean ± SD) | 15.19 ± 2.89 |
