# Supplementary material for: Early monitoring of the human polyomavirus BK replication and sequencing analysis in a cohort of adult kidney transplant patients treated with basiliximab
Source: Virol J. 2011 Aug 17;8:407. doi: 10.1186/1743-422X-8-407 (PMC3179958; doi:10.1186/1743-422X-8-407)
Supplement: Additional file 2 — BKV isolates analysed in this study. Table S2 includes all the BKV isolates analyzed in this study. [file 1743-422X-8-407-S2.DOC]

Table 2. *BKV isolates analysed in this study*

| Subtype/  Subgroup | Isolate | GenBank  Accession number | References |
| --- | --- | --- | --- |
| I/a | DUN | NC_001538 | Seif et al. (1979) |
| I/a | KEN-1 | AB263926 | Zheng et al. (2007) |
| I/a | CAF-5 | AB263913 | Zheng et al. (2007) |
| I/b-1 | Dik | AB211369 | Nishimoto et al. (2006) |
| I/b-1 | WW | AB211371 | Nishimoto et al. (2006) |
| I/b-1 | KOM-1 | AB211373 | Nishimoto et al. (2006) |
| I/b-2 | JL | AB211370 | Nishimoto et al. (2006) |
| I/b-2 | FNL-12 | AB263918 | Zheng et al. (2007) |
| I/b-2 | ITA-5 | AB263925 | Zheng et al. (2007) |
| I/c | MT | AB211372 | Nishimoto et al. (2006) |
| I/c | TW-1 | AB211381 | Nishimoto et al. (2006) |
| I/c | RYU-2 | AB211377 | Nishimoto et al. (2006) |
| I/c | KOM-6 | AB211375 | Nishimoto et al. (2006) |
| II | SB | Z19536 | Jin et al. (1993) |
| II | ETH-3 | AB263916 | Zheng et al. (2007) |
| II | GBR-12 | AB263920 | Zheng et al. (2007) |
| III | AS | M23122 | Tavis et al. (1989) |
| III | KOM-3 | AB211386 | Nishimoto et al. (2006) |
| IV/a-1 | VNM-7 | AB269869 | Nishimoto et al. (2007) |
| IV/a-1 | PHL-8 | AB269859 | Nishimoto et al. (2007) |
| IV/a-1 | MMR-24 | AB269842 | Nishimoto et al. (2007) |
| IV/a-1 | SEC-3 | AB269860 | Nishimoto et al. (2007) |
| IV/a-2 | RYU-3 | AB211389 | Nishimoto et al. (2006) |
| IV/a-2 | MMR-1 | AB269841 | Nishimoto et al. (2007) |
| IV/a-2 | FUJ-13 | AB269826 | Nishimoto et al. (2007) |
| IV/a-2 | VNM-2 | AB269868 | Nishimoto et al. (2007) |
| IV/b-1 | THK-8 | AB211390 | Nishimoto et al. (2006) |
| IV/b-1 | TW-3 | AB211391 | Nishimoto et al. (2006) |
| IV/b-1 | JPN-32 | AB269836 | Nishimoto et al. (2007) |
| IV/b-1 | JPN-33 | AB269837 | Nishimoto et al. (2007) |
| IV/b-2 | KOM-2 | AB211387 | Nishimoto et al. (2006) |
| IV/b-2 | JPN-15 | AB269834 | Nishimoto et al. (2007) |
| IV/b-2 | JPN-34 | AB269838 | Nishimoto et al. (2007) |
| IV/b-2 | MON-8 | AB269851 | Nishimoto et al. (2007) |
| IV/b-2 | KOM-7 | AB211388 | Nishimoto et al. (2006) |
| IV/b-2 | JPN-31 | AB269835 | Nishimoto et al. (2007) |
| IV/c-1 | MON-1 | AB269846 | Nishimoto et al. (2007) |
| IV/c-1 | SWC-1 | AB269863 | Nishimoto et al. (2007) |
| IV/c-1 | NEC-4 | AB269854 | Nishimoto et al. (2007) |
| IV/c-1 | NWC-8 | AB269858 | Nishimoto et al. (2007) |
| IV/c-1 | MMR-28 | AB269843 | Nishimoto et al. (2007) |
| IV/c-1 | SEC-6 | AB269861 | Nishimoto et al. (2007) |
| IV/c-1 | NWC-14 | AB269855 | Nishimoto et al. (2007) |
| IV/c-1 | FUJ-18 | AB269827 | Nishimoto et al. (2007) |
| IV/c-1 | VNM-1 | AB269867 | Nishimoto et al. (2007) |
| IV/c-2 | GRC-4 | AB269830 | Nishimoto et al. (2007) |
| IV/c-2 | ITA-4 | AB269833 | Nishimoto et al. (2007) |
| IV/c-2 | SWE-4 | AB269866 | Nishimoto et al. (2007) |
| IV/c-2 | FIN-2 | AB269822 | Nishimoto et al. (2007) |
| IV/c-2 | MON-5 | AB269849 | Nishimoto et al. (2007) |
