# Supplementary material for: Early monitoring of the human polyomavirus BK replication and sequencing analysis in a cohort of adult kidney transplant patients treated with basiliximab
Source: Virol J. 2011 Aug 17;8:407. doi: 10.1186/1743-422X-8-407 (PMC3179958; doi:10.1186/1743-422X-8-407)
Supplement: Additional file 3 — BKV detection in plasma and urine with relative viral load in recipients at different times. Table S3 shows the BK viral loads detected in plasma and urine of renal transplant recipients. [file 1743-422X-8-407-S3.DOC]

*Table 3. BKV detection in plasma and urine with relative viral load in recipients at d*ifferent times.

| *Pharmacokinetic of basiliximab* | *Samples collection time* | *Samples* | | | | | | | | | |
| --- | --- | --- | --- | --- | --- | --- | --- | --- | --- | --- | --- |
|  |  | PLASMA | | | | | URINE | | | | |
|  |  | Pos (%) | Neg (%) | *p** | Viral load, median (range) log GEq/ml | *p*** | Pos (%) | Neg (%) | *p** | Viral load, median (range) log GEq/ml | *p*** |
| *1st dose*  *(day 0)* | *Tx* | 16 (26) | 44 (74) | 0,550 | 3,79  (3,26 - 4,06) | 0,255 | 4 (6) | 56 (94) | 0,155 | 3,70  (2,85 - 4,04) | 0,001 |
| *2nd dose*  *(day 4)* | *T1* | 20 (33) | 40 (67) |  | 4,00  (3,22 - 4,32) |  | 10 (16) | 50 (84) |  | 4,40  (3,97 - 5,62) |  |
|  |  |  |  | 0,043 |  | << 0,05 |  |  | << 0,05 |  | << 0,05 |
| *IL-2Rα saturation*  *(day 59)* | *T2* | 32 (53) | 28 (47) |  | 4,52  (4,25 - 4,71) |  | 32 (53) | 28 (47) |  | 5,78  (5,67 - 5,94) |  |
|  |  |  |  | 0,009 |  | << 0,05 |  |  | 0,009 |  | << 0,05 |
| *Manteinance therapy only* | *T3* | 17 (28) | 43 (72) |  | 4,00  (3,43 - 4,29) |  | 17 (28) | 43 (72) |  | 4,09  (3,58 - 4,32) |  |

NOTE: GEq: genome equivalents

* By χ^2^  test

** By Student’s t-test

*Tx =* 12 hours post-transplantation; *T1 =* 1 month post-transplantation; *T2 =* 3 months post-transplantation; *T3 =* 6 months post-transplantation

*1st dose (day 0):* intravenous administration of the first 20 mg of basiliximab within 2 hours prior to transplantation surgery

*2nd dose (day 4):* intravenous administration of the second 20 mg of basiliximab 4 days after transplantation

*IL-2Ra saturation:* MMF-based triple immunotherapy significantly increases the duration of IL-2Ra saturation (36 vs 59 days)

*Manteinance therapy:* tacrolimus (or cyclosporine), MMF and corticosteroids
